# Sublethal application of various sulfonylurea and imidazolinone herbicides favors outcrossing and hybrid seed production in oilseed rape

**DOI:** 10.1186/s12870-020-2278-9

**Published:** 2020-02-11

**Authors:** Cheng-Yu Yu, Jing-long Lian, Qiong Gong, Li-Suo Ren, Zhen Huang, Ai-Xia Xu, Jun-Gang Dong

**Affiliations:** College of Agronomy, Northwest A&F University, Yangling, 712100 Shaanxi China

**Keywords:** *Brassica napus*, Male sterility, Gametocide, Chemical hybridizing agents, Herbicide, Sulfonylurea, Imidazolinone

## Abstract

**Background:**

Acetolactate synthase (ALS)-inhibiting herbicides from the chemical families of sulfonylureas and imidazolinones are used worldwide. However, drift or sprayer contamination from some sulfonylurea herbicides causes a high level of male sterility in cruciferous species, especially oilseed rape (OSR). In this paper, we evaluated the gametocidal effects of 27 ALS-inhibiting herbicides that were sprayed on OSR plants at the bolting stage.

**Results:**

OSR anther development was very sensitive to sublethal exposure to most ALS-inhibiting herbicides. The application of 18 out of the 20 tested sulfonylureas (except ethametsulfuron and ethoxysulfuron), two imidazolinones (imazethapyr and imazamox), and one sulfonylamino-carbonyltriazolinone (flucarbazone-sodium) at suitable rates could induce male sterility. Eight of the herbicides, including chlorsulfuron (at application rates of 60–120 mg/ha), halosulfuron-methyl (300–600 mg/ha), sulfosulfuron (400–600 mg/ha), triflusulfuron-methyl (500–750 mg/ha), pyrazosulfuron-ethyl (150–225 mg/ha), nicosulfuron (200–300 mg/ha), imazethapyr (750–1125 mg/ha), and imazamox (400–800 mg/ha), could induce over 90% male sterility and over 60% relative outcrossed seed set in six cultivars with different origins. These eight chemicals could be used as new gametocides for hybrid seed production. This study also examined the possibility of external application of these gametocides on several unstable Polima cytoplasmic male sterile and thermosensitive genic male sterile lines. Although the outcrossed seed set of the treated lines was slightly reduced, the gametocide application significantly increased the seed purity of the resulting hybrid.

**Conclusion:**

The finding of the gametocidal effects of most sulfonylureas and imidazolinones are of great importance for developing new functions for ALS-inhibiting herbicides. The application of gametocides will also greatly promote the safe utilization of environment-sensitive male sterility in hybrid seed production. Unexpectedly, the application of three triazolopyrimidines (florasulam, flumetsulam, and penoxsulam) and one pyrimidinylthiobenzoate (bispyribac-sodium) did not cause male sterility, although these herbicides obviously inhibited the activity of ALS and plant growth. This result suggests that inhibition of ALS activity does not always lead to male sterility in plants, and these gametocides may also inhibit other biological functions vital for microspore development.

## Background

The large-scale adoption of hybrid oilseed rape (OSR, *Brassica napus*) contributes significantly to the oilseed supply worldwide. To economically produce hybrid seeds on a large scale, an effective male sterility (MS) system is crucial to reduce selfing and ensure outcrossing in the female parent because OSR is a predominantly self-pollinated crop [1]. At least seven MS systems are deployed for hybrid production in OSR, but all have some defects. First, many inheritable MS systems have been shown to be associated with some adverse effects on disease resistance, oil content, or other biological traits [2–8]. Second, it is time-consuming and genotypically dependent to develop various male-sterile, maintainer, and restorer lines for cytoplasmic male sterile (CMS) and genic MS (GMS) systems. Third, there is great risk in applying Polima CMS [9] and thermosensitive male sterility (TMS) [10] in hybrid seed production in the world’s second-highest OSR producer, China, because CMS and TMS are unstable when the temperature before flowering changes greatly [8, 10, 11].

Chemical induction of male sterility (CIMS) by the use of gametocides/chemical hybridizing agents is an important method for hybrid seed production because the method avoids the problems of adverse effects from MS genes and saves the considerable time spent on prebreeding in the development of MS lines. The possible use of CIMS has been investigated in all major crops [12], but the use of many gametocides, for example, Genesis (clofencet) for wheat hybridization [13], has ceased owing to the high cost of gametocides, low seed production, or high risk of low seed purity (hybridity). However, rich experience in the utilization of CIMS has been accumulated for hybrid OSR, mainly in China. In recent decades, researchers have developed various types of gametocides for OSR, such as ethrel/ethephon [14], gibberellins, DPX-3773 [15], methyl arsenate [16], benzotriazole [17], and the synthetic detergent Surf Excel [18]. Unfortunately, to our knowledge, most of these gametocides are not practical for commercial use due to market availability, high pollution [16], or low efficacy. From this view, the development of effective and relatively cheap gametocides without environmental or health risks is very important for utilizing the heterosis of two-line hybrids in OSR.

It was found that the application of some sulfonylurea (SU) herbicides, such as tribenuron-methyl (TBM) [19] and amidosulfuron [20], had a strong gametocidal effect on OSR and allowed the treated plants to outcross. These SUs have been validated as useful gametocides for OSR and other *Brassica* species, and now, their different forms have been widely used by many institutes and seed companies in China [21]. More than 20 commercial hybrids based on SU gametocides have been released in China [21] by OSR breeders from Shaanxi, Hubei, Chongqing, Hunan, Sichuan, Jiangsu, Zhejiang, and Jiangxi provinces. SU herbicides target the acetolactate/acetohydroxylate synthase (ALS/AHAS) enzyme, which is a key enzyme in the biosynthesis of valine, leucine, and isoleucine. There are hundreds of chemicals targeting ALS, including the five main families of SU, imidazolinone (IM), triazolopyrimidine (TP), pyrimidinylbenzoate (PB), and sulfonylamino-carbonyltriazolinone (SC) [22]. Species from the Brassica family are very sensitive to most ALS-inhibiting herbicides, and either herbicide drift or sprayer contamination can cause phytotoxicity [23]. Thus, we pose a question: is it feasible to screen some new gametocides from these herbicides?

In the present paper, 27 ALS-inhibiting herbicides were sprayed on OSR at sublethal doses to compare the effects of the herbicides on the reproductive tissues. We also evaluated the seed quality and hybrid purity of some hybrid production trials using several selected herbicides. Another objective of the study was to achieve stable pollen sterility in several temperature-sensitive lines of CMS and TMS by gametocide application. The results are useful for evaluating the influences of herbicides on the reproduction of OSR and the development of new gametocides for OSR or other Brassica species.

## Results

### The gametocidal effect of herbicides on *Brassica napus*

**Fig. 1:**
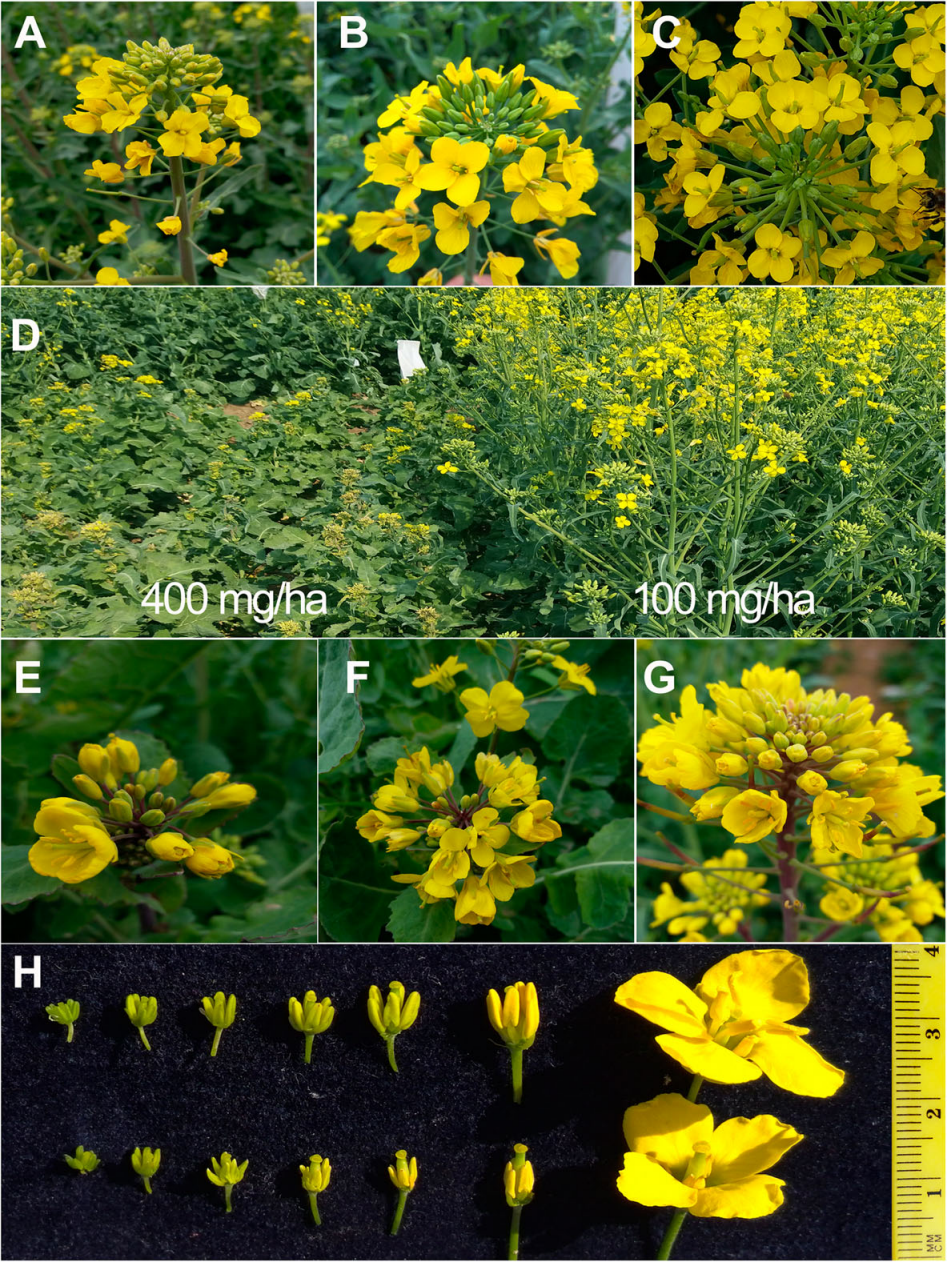
Flowers of OSR plants exposed to sublethal doses of different herbicides. Completely sterile flowers caused by application of 750 mg/ha imazethapyr (**a**), 120 mg/ha chlorsulfuron (**b**), and 500 mg/ha triflusulfuron-methyl (**c**). Too high of doses of nicosulfuron (400 mg/ha) caused serious injury (left), but the 100 mg/ha treatment (right) resulted only in male sterility (**d**). The application of 6000 mg/ha bispyribac-sodium (**e**), 600 mg/ha flumetsulam (**f**), and 270 mg/ha ethoxysulfuron (**g**) did not cause male sterility in the anthers of the flower buds at different developmental stages. Comparison of the anther and filament size of the fertile (upper) and sterile (lower) flowers, where the male sterility was caused by chlorsulfuron (**h**)

Among the 27 different herbicides that were used in this experiment, 21 herbicides showed different gametocidal effects on the OSR cultivar Qin8C (Table 1). Application of 18 out of the 20 SUs (except ethametsulfuron and ethoxysulfuron) and two IMs, imazethapyr (Fig. 1a) and imazamox, at their suitable dose induced obvious MS in *B. napus*. The effect of the designed dose for the SC member flucarbazone-sodium was not ideal owing to the lower than 80% induction rate of MS plants (Table 1). Twelve of the SUs, namely, bensulfuron, chlorimuron-ethyl, chlorsulfuron (Fig. 1b), halosulfuron-methyl, flazasulfuron, metsulfuron-methyl, monosulfuron, nicosulfuron, oxasulfuron, pyrazosulfuron-ethyl, sulfosulfuron, and triflusulfuron-methyl (Fig. 1c), induced MS in more than 90% of the treated plants and lower damage in their suitable dose range. Too high of doses resulted in obvious pesticide damage, for example, growth stop and plant death (Fig. 1d).

**Table 1:** Gametocidal effect of sublethal dose application of various herbicides on OSR cv. Qin8C^a^

Herbicide	Dosage (mg ha^-1)^	Percentage of MS plant (%)	SD	Percentage of damaged plant (%)	SD
Bensulfuron	400	100.0	0.0	96.5	6.0
	300	99.9	0.2	27.8	5.1
	200	**91.7**	3.3	**0.0**	0.0
	100	13.5	6.4	0.0	0.0
Chlorimuron-ethyl	360	100.0	0.0	97.9	2.3
	240	**100.0**	0.1	**12.1**	3.6
	120	86.2	2.6	0.0	0.0
Chlorsulfuron	240	100.0	0.0	95.3	5.3
	180	100.0	0.0	49.3	6.9
	120	**100.0**	0.0	**11.1**	1.4
	60	**90.4**	4.3	**1.6**	1.6
Ethoxysulfuron	405	0.0	0.0	96.9	5.4
	270	0.0	0.0	26.4	6.1
	135	0.0	0.0	0.0	0.0
Ethametsulfuron	60,000	1.9	1.6	19.4	2.7
	40,000	0.0	0.0	0.0	0.0
	20,000	0.0	0.0	0.0	0.0
Flazasulfuron	675	100.0	0.0	72.1	2.7
	450	79.8	2.6	38.0	1.7
	225	41.3	5.4	0.0	0.0
Foramsulfuron	450	100.0	0.0	93.3	5.9
	300	86.6	6.4	37.0	3.7
	150	2.4	0.5	0.0	0.0
Halosulfuron-methyl	1200	100.0	0.0	100.0	0.0
	900	100.0	0.0	73.4	6.3
	600	**99.9**	0.1	**3.4**	1.9
	300	**89.8**	2.6	**0.0**	0.0
Idosulfuron-methyl sodium	180	100.0	0.0	62.0	12.9
	120	70.7	4.4	33.0	5.3
	60	23.2	3.9	0.0	0.0
Mesosulfuron-methyl	450	100.0	0.0	95.9	6.3
	300	60.2	10.9	48.3	11.2
	150	37.1	3.3	0.0	0.0
Metsulfuron-methyl	360	100.0	0.0	95.6	7.6
	240	**99.9**	0.2	**17.2**	3.0
	120	56.2	9.8	13.3	6.7
Monosulfuron	400	100.0	0.0	40.0	2.8
	300	**100.0**	0.0	**14.6**	7.9
	200	75.7	5.2	1.3	1.2
	100	14.7	4.8	0.0	0.0
Nicosulfuron	400	100.0	0.0	49.4	12.1
	300	**100.0**	0.0	**10.7**	4.3
	200	**92.8**	2.4	**0.0**	0.0
	100	61.2	5.3	0.0	0.0
Oxasulfuron	1200	100.0	0.0	98.1	3.3
	900	100.0	0.0	34.9	9.5
	600	**99.8**	0.2	**4.4**	1.9
	300	62.8	4.9	0.0	0.0
Pyrazosulfuron-ethyl	300	100.0	0.0	73.1	10.8
	225	**100.0**	0.0	**20.3**	8.0
	150	**91.9**	3.0	**9.2**	2.4
	75	27.8	4.9	0.9	0.8
Rimsulfuron	600	100.0	0.0	100.0	0.0
	400	71.2	11.4	79.0	5.1
	200	44.4	4.6	9.9	5.4
Sulfometuron-methyl	2200	100.0	0.0	91.6	7.4
	1650	100.0	0.0	45.1	4.5
	1100	72.3	3.8	0.9	0.7
	550	22.4	3.1	1.0	0.9
Sulfosulfuron	800	100.0	0.0	89.4	7.5
	600	**100.0**	0.0	**18.7**	7.2
	400	**99.9**	0.1	**7.8**	5.0
	200	82.1	2.4	0.9	0.0
Thifensulfuron	300	100.0	0.0	93.4	2.9
	200	77.3	5.1	36.6	2.9
	100	30.4	8.7	0.0	0.0
Triflusulfuron-methyl	1000	100.0	0.0	60.0	4.6
	750	**100.0**	0.0	**1.4**	0.8
	500	**100.0**	0.0	**0.0**	0.0
	250	31.7	8.9	0.0	0.0
Imazethapyr	1500	100.0	0.0	33.7	5.6
	1125	**100.0**	0.0	**17.8**	4.2
	750	**94.6**	4.9	**0.6**	1.1
	375	33.6	9.7	0.0	0.0
Imazamox	800	100.0	0.0	56.8	6.9
	600	**100.0**	0.0	**11.7**	3.1
	400	**95.3**	3.6	**0.0**	0.0
	200	30.4	5.4	0.0	0.0
Florasulam	600	0.0	0.0	96.7	2.9
	400	0.0	0.0	33.8	7.8
	200	0.0	0.0	0.0	0.0
Flumetsulam	600	0.0	0.0	100.0	0.0
	400	0.0	0.0	21.6	8.1
	200	0.0	0.0	0.0	0.0
Penoxsulam	600	0.0	0.0	100.0	0.0
	450	0.0	0.0	20.3	5.4
	300	0.0	0.0	2.7	2.2
	150	0.0	0.0	0.0	0.0
Bispyribac-sodium	800	0.0	0.0	100.0	0.0
	600	0.0	0.0	78.7	5.7
	400	0.0	0.0	29.4	8.0
	200	0.0	0.0	12.6	2.3
Flucarbazone-sodium	300	90.2	5.4	70.4	15.9
	200	62.4	8.6	1.9	1.4
	100	5.7	1.2	0.0	0.0

^a^Values in bold type show the treatment resulting in both satisfactory gametocidal effect and acceptable phytotoxicity. *SD* means standard deviation

**Fig. 2:**
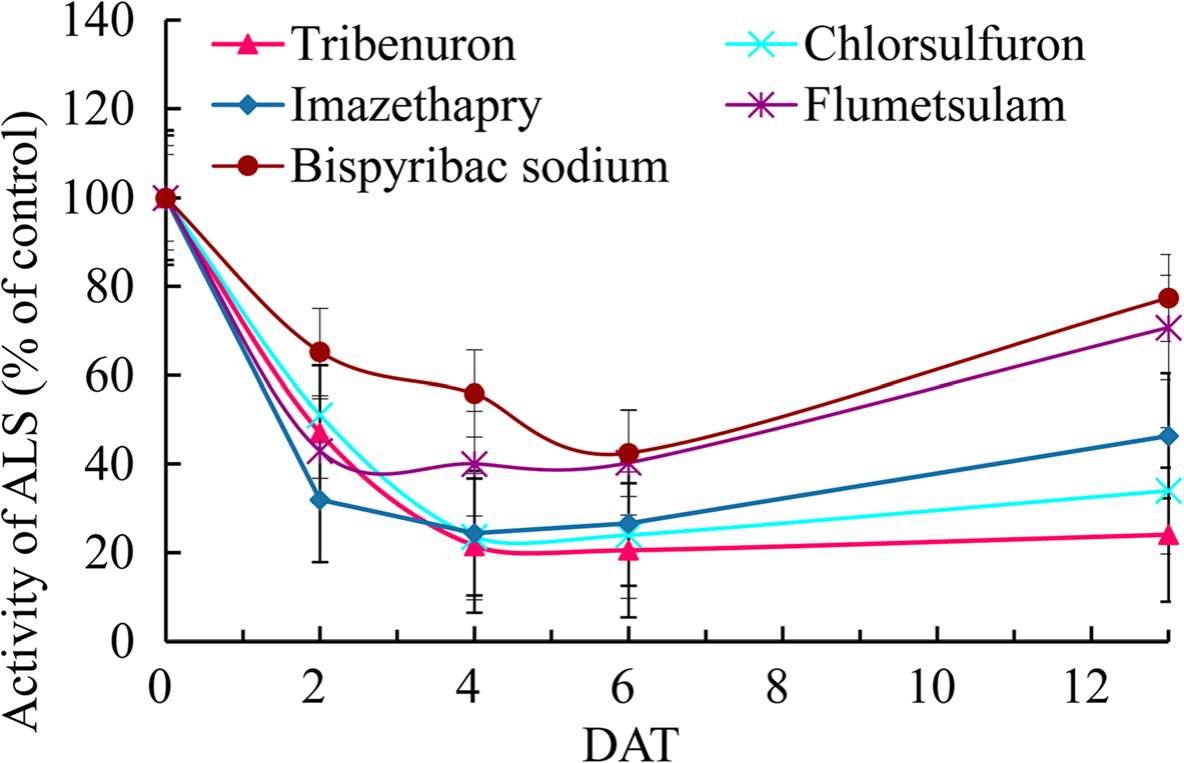
Inhibition of the in vivo ALS activity of OSR flower buds by several herbicides. Treatments: tribenuron-methyl (60 mg/ha), chlorsulfuron (60 mg/ha), imazethapyr (750 mg/ha), bispyribac-sodium (6000 mg/ha), flumetsulam (600 mg/ha). DAT: days after treatment

The three TPs (florasulam, flumetsulam, and penoxsulam) and one PB (bispyribac-sodium) tested did not cause MS, although higher doses of these herbicides caused obvious plant injury, including severe leaf variegation (Fig. 1e-g) and temporal stop of stem node elongation, as did higher doses of ethoxysulfuron. We assayed the in vivo ALS activity and found that these herbicides also obviously inhibited the activity of the ALS enzyme, as did the SU herbicides, but the inhibition lasted only for times shorter than those from TBM and amidosulfuron (Fig. 2).

### The phenotypic variations caused by ALS inhibitors

The development of both the vegetative and reproductive parts of OSR was suppressed by most of the treatments, and the higher doses resulted in a more obvious suppression effect (Table 2). The male parts of the flower bud, the filaments and anthers, were more impacted by these gametocidal treatments (Table 2). At the early growth stage (bud length ≤ 3 mm), no obvious difference was found visually in the flower buds of the plants treated with 100 mg/ha chlorsulfuron. After the uninucleate stage (flower bud length 3–3.5 mm), the elongation of the filaments and the expansion of the anthers were inhibited, and ultimately, all the anthers withered before blooming (Fig. 1h). In contrast, the female part, the pistil, was not severely affected (Fig. 1h). These results showed that the stamen development of OSR is more sensitive to ALS-inhibiting gametocides than that of the pistil.

**Table 2:** Effect of chlorsulfuron treatments on several biological traits of OSR cv. Qin8C^a^

Dose (mg/ha)	Petal width (mm)	Petal length (mm)	Pistil length (mm)	Delay days of first flower open	Duration of male sterility (day)	Duration of flowering on terminal raceme (day)	Plant height (cm)
240	7.87B	9.33C	10.81B	3.05A	16.05B	11.40B	109.91B
180	8.99A	10.10B	11.36AB	2.05B	21.10A	19.05A	154.20A
120	9.04A	10.50B	11.54AB	0.70C	17.20B	20.20A	165.82A
0	9.21A	11.41A	11.79A	0.35C	0.00C	20.00A	164.96A

^a^Letters after data indicate significant differences at 0.01 level

In addition to MS, all treatments at higher doses caused stunting of growth, chlorosis in leaves, and fading in flower buds in the first few days after treatment. The degree of plant injury depended on the application rate of herbicides, and injury was more evident for the treatments with higher doses. For example, the slight chlorosis in the leaves caused by an application dose of 120 mg/ha of chlorsulfuron recovered in three to 5 days, but the recovery after an application dose of 240 mg/ha required more than 1 week. The height of the plants treated with chlorsulfuron at an application dose of 240 mg/ha decreased, the beginning of flowering was delayed by two to 5 days, and the duration of flowering on the main inflorescence was shortened. The length of the pistil, petal size, and flowering duration were also reduced by higher doses (Table 2).

### Validation of the gametocidal activity of eight selected herbicides

All eight selected herbicides (chlorsulfuron, halosulfuron-methyl, nicosulfuron, pyrazosulfuron-ethyl, sulfosulfuron, triflusulfuron-methyl, imazethapyr, and imazamox) showed strong gametocidal effects, comparable to the standard gametocide TBM (Table 3). Exposure to these herbicides resulted in almost 0% selfed seed set and over 60% relative outcrossed seed set in comparison to the control. The lower application doses led to moderate phytotoxicity (Table 3). The higher application doses resulted in higher MS, but the consequent problem of pesticide damage resulted in shortened inflorescence and poor seed set (Table 3). For example, seed yields under open pollination from the application of 60 and 120 mg/ha chlorsulfuron were 83.65 and 61.31% that of the control, respectively. The reduction in outcrossed seed yield observed in most treatments was due to the reductions in both the number and length of siliques.

**Table 3:** Average effects of nine gametocides on the fertility of six *Brassica napus* cultivars

Chemical	Dose mg/ha	Percentage of male sterile plant	SD	Self-pollinated seed-setting rate	SD	Seed-setting rate under open pollination	SD
Chlorsulfuron	60	97.15	3.62	0.05	0.19	83.65	7.77
Chlorsulfuron	120	100.00	0.00	0.00	0.00	61.31	8.38
Halosulfuron methyl	300	98.09	2.69	1.17	1.07	72.70	10.97
Halosulfuron methyl	600	100.00	0.00	0.00	0.00	46.15	8.98
Sulfosulfuron	400	99.40	1.44	0.00	0.00	86.87	6.47
Sulfosulfuron	600	100.00	0.00	0.00	0.00	59.92	11.09
Triflulsulfuron-methyl	500	96.97	3.09	0.88	0.65	90.11	7.84
Triflulsulfuron-methyl	750	100.00	0.00	0.00	0.02	76.81	8.27
Nicosulfuron	200	98.95	2.07	0.00	0.01	78.22	8.12
Nicosulfuron	300	100.00	0.00	0.00	0.00	68.70	9.02
Pyrazosulfuron-ethyl	150	89.73	4.89	5.36	4.42	87.06	20.58
Pyrazosulfuron-ethyl	225	99.90	0.43	0.00	0.00	62.00	7.76
Imazethapyr	750	90.60	3.05	2.15	1.27	98.21	9.48
Imazethapyr	1125	99.79	0.62	0.00	0.00	74.52	8.72
Imazamox	400	93.13	5.08	1.53	1.61	105.20	10.00
Imazamox	600	98.98	1.98	0.00	0.02	86.46	6.39
TBM	60	99.71	0.84	0.01	0.02	84.02	7.57
TBM	120	100.00	0.00	0.00	0.00	65.52	8.09
Control	0.0	0.00	0.00	100.00	0.00	100.00	0.00

### The response of different OSR genotypes to gametocides

**Fig. 3:**
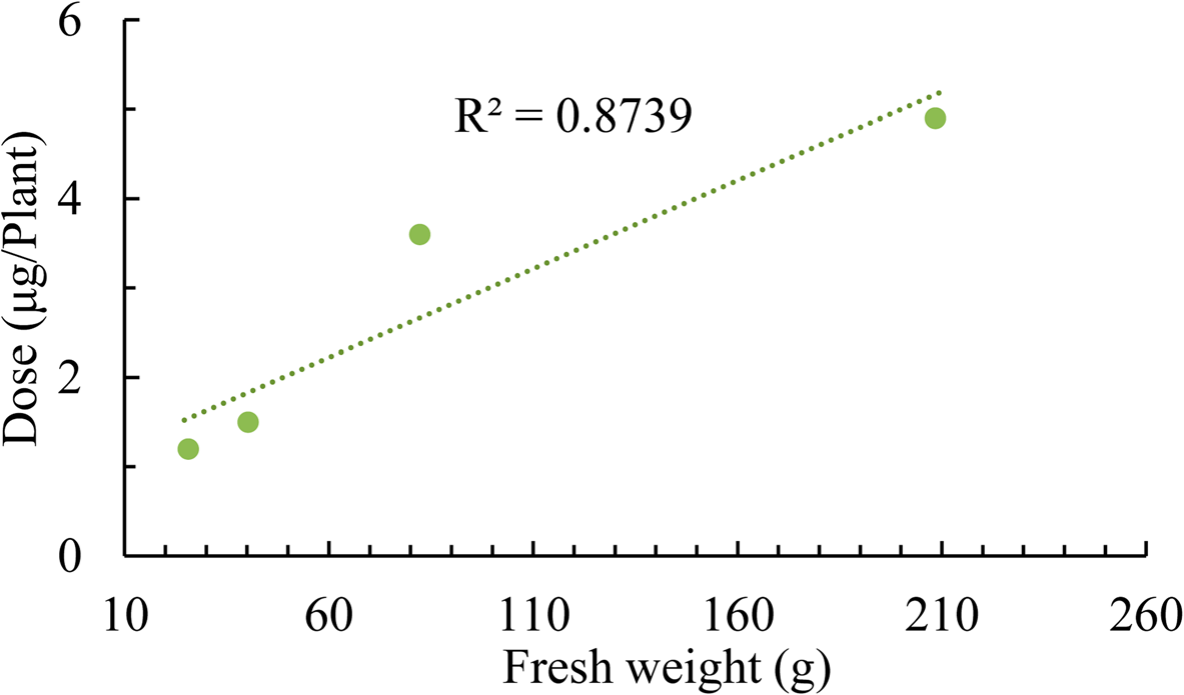
Relationships between the optimal dose of chlorsulfuron with the fresh weight (g) of cultivar M267

The results for analysis of variance of the field experiment in the split-plot design (Additional file 1: Table S2) showed that the gametocidal effects (percentage of MS plant) and phytotoxicity (open-pollination seed-set rate) on the six cultivars (main plots in the field experiment), treatment effects of the two doses (subplots) of the nine gametocides, and the interaction between the genotypes and gametocide treatments were significant. The differences among the selfed seed-set rates of different gametocide treatments were also significant. We found that the gametocidal effect had a genotype preference, and the cultivars with vigor seedlings (H15 and M267) needed a higher dose than cultivars with weak seedlings (Zhong9 and Zhe18). There was an obvious association between treatment with the optimal dose and reduced plant biomass in four batch seedlings of cultivar M267, whose seeds were sown in batches at five-day intervals (Fig. 3).

### Seed set of small-scale trials for hybrid production

A total of 6.05 kg and 8.38 kg of hybrid seeds were collected from the two isolated cages for the biparental crosses of Zhong9 CIMS × BC530 and Q5005 CIMS × BC530, which were 18.78% lower and 0.85% higher than their corresponding Polima CMS lines, respectively. The thousand-grain weight and germination rate of the seeds were slightly and not significantly lower, respectively, than those of the hybrid seeds from their corresponding CMS lines due to many unfilled seeds (Table 4). The decreases in seed yield, seed weight and germination rate intimated either impairment of the seed development or a decrease in the silique number of the CIMS plants. The true hybrid seedlings resembled the male parent BC530 in the trait of deep-divided leaves and thus could be easily distinguished from the undesirable plants derived from female parent selfing. The average hybridity (hybrid seed percentage) of the seeds collected from Zhong9 CIMS × BC530 and Q5005 CIMS × BC530 were 93.0 and 91.4% (Table 4), respectively, which was higher than the national standard of 85% for the hybridity of OSR hybrid seed lots.

**Table 4:** The quality of seeds obtained from the plants exposed to chlorsulfuron

Seed samples	Seed yield (g/plant)	1000-grain-weight (g)^a^	Germination rate (%)^a^	Hybridity (%)^b^
Zhong9 CIMS× BC530	13.75 ± 0.66**	3.52 ± 0.08	90.7 ± 3.10	93.0 ± 1.87
Zhong9-A× BC530	16.93 ± 0.95	3.74 ± 0.08	97.2 ± 2.23	96.7 ± 2.27
Q5005 CIMS× BC530	19.05 ± 0.94	3.86 ± 0.08	92.5 ± 2.10	91.4 ± 2.80
Q5005-A× BC530	18.89 ± 0.83	3.98 ± 0.07	96.8 ± 2.00	92.8 ± 2.53

^a^The coarse seed lot contained unfilled seeds

^b^The seed lot experienced cleaning process and contained only plump seeds

The symbol ** indicates significant difference at 0.01 level

### The effect of gametocide on eliminating pollen in CMS and TMS lines

**Fig. 4:**
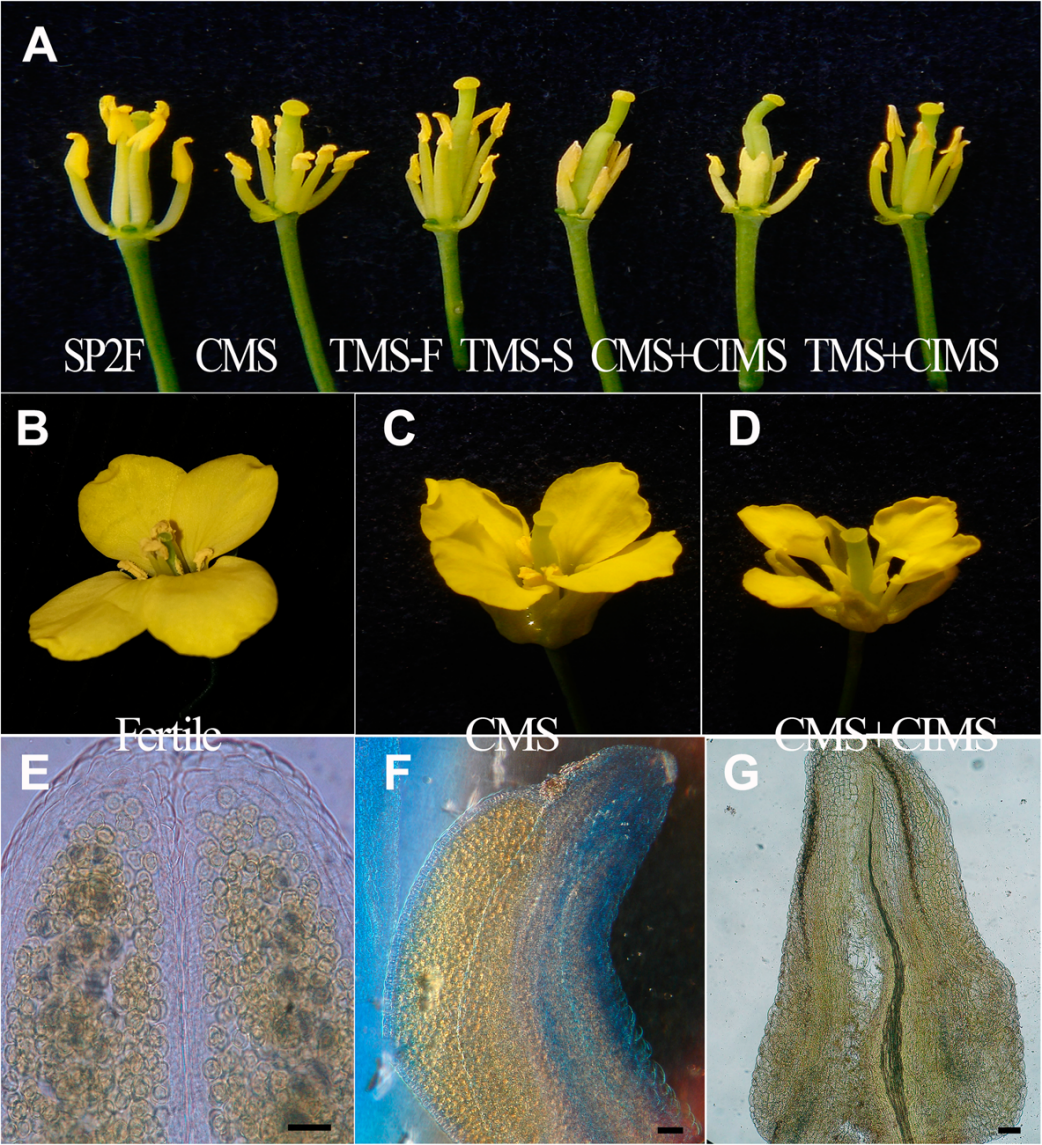
Phenotypic comparison of different flowers. **a** The stamens of maintainer line SP2, CMS SP2-A, TMS SP2S-under low temperature, TMS SP2S-under high temperature, CMS SP2-A + CIMS, and TMS SP2S + CIMS. The flowers of maintainer line SP2 (**b**, fertile), CMS SP2-A (**c**, partially sterile), and CMS SP2-A + CIMS (**d**, sterile). Pollen grains in the anthers of maintainer line SP2 (**e**, fertile), CMS SP2-A (**f**, partially sterile), and CMS SP2-A + CIMS (**g**, sterile). Scale bar = 50 μm

In the first week of flowering in this experimental season, the pollen viability of the CMS lines Zheyou50-A, Q5005-A, SP2-A, and TMS SP2S was very high (Table 5). This defect prevents their safe utilization in hybrid production. Exposure of these lines to chlorsulfuron at a 100 mg/ha rate resulted in nearly 100% MS in the population and over the whole flowering period (Table 5). The stamens of CMS SP2-A + CIMS and TMS SP2S + CIMS were much smaller than those of the fertile line SP2, partially sterile CMS SP2-A, and fertile phase of SP2S TMS at low temperature (Fig. 4a). Compared to the flowers of the fertile maintainer line SP2 (Fig. 4b), CMS SP2-A was partially sterile (Fig. 4c), and CMS SP2-A treated by chlorsulfuron (CMS + CIMS) was completely sterile (Fig. 4d). The differences were notable when the pollen viability was examined under a microscope. The fertile anthers contained plentiful round pollen grains (Fig. 4e). Polima CMS line SP2-A presented partial MS because one or two of the four locules in the anther swelled and produced some pollen grains, leading to a crescent anther (Fig. 4f). However, the aborted anthers from CMS + CIMS were undeveloped, containing no pollen (Fig. 4g) or only a few distorted pollen shells.

**Table 5:** Effect of application of 100 mg/ha chlorsulfuron on environment sensitive male-sterile lines

Male sterile line	Gametocide treatment	Pollen viability at early flowering stage %	SD	Seed-set of out-crossing (g/plant)^c^	SD	Hybridity of samples %^c^	SD
Zhong9-A	untreated	5.46	4.38	17.79	1.10	97.50	1.55
	treated	0.01^b^	0.04	13.08^b^	1.06	100.00	0.00
Q5005-A	untreated	10.08	4.31	20.19	1.08	92.50	5.06
	treated	0.05^b^	0.07	20.75	1.86	98.69	1.88
Sapphire-A	untreated	2.07	2.07	19.37	1.28	96.40	1.82
	treated	0.00	0.00	16.91^b^	1.20	100.00	0.00
Zheyou50-A	untreated	17.20	6.28	21.46	1.12	90.13	3.40
	treated	0.00^b^	0.00	20.73	0.74	99.73^a^	0.47
SP2-A	untreated	30.21	14.47	22.82	1.51	85.85	3.99
	treated	0.00^b^	0.01	20.35^b^	1.24	98.52^b^	1.22
SP2S	untreated	59.51	10.45	20.53	1.72	96.88	2.39
	treated	0.01^b^	0.03	19.03	1.53	100.00	0.00

The symbol ^a^ and ^b^ indicate significant difference at 0.05 and 0.01 level respectively. ^c^ The plants were pollinated by the paternal line BC530

Pistil function was also somewhat affected by gametocide application. The outcrossed seed set of chlorsulfuron-treated plants of CMS lines, including Zhong9-A, Sapphire-A, Zheyou50-A, SP2-A, and TMS line SP2S, decreased by 26.5, 12.7, 3.4, 10.8, and 7.3% compared with their untreated controls, respectively, with an exceptional increase of 2.8% in Q5005-A. Although the seed set was depressed, the self-pollination was constrained well, as evidenced by almost no selfed seed set under bagged conditions and a high level of seed hybridity under outcrossed conditions (Table 5). Other gametocide treatments, such as triflusulfuron-methyl, nicosulfuron, and imazethapyr, could also induce a significantly high percentage of MS in the treated plants of CMS and TMS lines (unpublished data).

## Discussion

### SUs and IMs will be substantial chemical sources for the selection of new gametocides

Most SU herbicides can strongly inhibit the growth of weeds and volunteer crops, and some of them also have good gametocidal effects on OSR and other sensitive plants [20]. This effect cannot be ignored in the evaluation of herbicide damage and the safe use of ALS-inhibiting herbicides. A gametocide is rather difficult to invent because it must have strong positional selectivity between the stamen and pistil. The well-known herbicide glyphosate caused transient MS in Brassica [24], but we found that it caused severe damage to the pistil and leaf (unpublished). Our results suggested that it is practical to screen gametocides from ALS-inhibiting herbicides, especially SUs and IMs, because exposure of different OSR cultivars to the eight selected gametocides resulted in a high degree of MS as well as lower phytotoxicity on the pistil. At present, more than 50 SU and IM herbicides have been commercialized, and small changes in the substituents may result in significant changes in their biological activity and species selectivity. There are thousands of compounds with the basic structures of SU, IM, TP, etc., and these substances provide a large bank for screening new gametocides. Limited by the experimental scale, we did not validate the gametocidal effects of the rest of the herbicides, including bensulfuron, chlorimuron-ethyl, metsulfuron-methyl, monosulfuron, and oxasulfuron. We believe that some of these herbicides may also be useful gametocides if more doses are tested for gametocidal treatment.

### Gametocidal effects are affected by various factors

We found that the TPs (florasulam, flumetsulam, and penoxsulam) and PB (bispyribac-sodium) tested did not cause MS, although these herbicides obviously inhibited the activity of the ALS enzyme. This result suggests that inhibition of ALS activity is not a guarantee of MS, as previous studies have suggested [25], and SU gametocides may also affect some other biological functions necessary for microspore development, such as plastid structure, tissue autophagy, ethylene release, detoxification, fatty acid metabolism, and cell cycles [26, 27]. These pathways would also be interesting for study with respect to the modes of action of SU and IM gametocides.

The existence of a genotypic effect from the gametocides in this study suggests that minor adjustment of the application dose is needed when different OSR cultivars are used as female parents for hybrid production. Several *ALS* gene mutations were found to be strongly tolerant of SUs [28–30], and these mutations in the male parent can be used in hybrid seed production based on CIMS [28]. In most cases, genotypic susceptibility is not a consequence of *ALS* gene mutation but from stems from some physiological aspects, such as absorption, transport, biological detoxification and defense responses [26, 27]. In our experience, some OSR cultivars with traits such as high growth speed, large biomass, thick wax powder, and long duration of flowering will need a higher application dose of SU gametocide. In addition to plant genotypic differences, other factors, including plant uniformity, plant developmental stage, climate conditions, adjuvants, and sprayer equipment, also influence the gametocidal effect.

### Application of gametocide to enhance the effectiveness of OSR breeding

The application of SU and IM gametocides can greatly enhance the effectiveness of OSR breeding by simplifying hybrid breeding programs, among other uses. Researchers attempt to use chemicals to kill pollen in environment-sensitive MS lines of OSR and other crops, such as rice TMS [31], to prevent the risk of fertility fluctuations in hybrid production systems. Our results showed that it is possible to achieve 100% MS in CMS or TMS lines with external application of some SU gametocides. Thus, the application of the double MS systems called ‘CMS + CIMS’ and ‘TMS + CIMS’ will greatly diminish the risk of seed production based on environment-sensitive MS. Moreover, because SU and IM gametocides can elicit nonheritable MS in plants, this technique will not only allow the production of hybrid seeds from large numbers of intervarietal and even interspecific crosses but also allow the production of many types of varieties in addition to single cross hybrids, such as reciprocal cross, triple-cross and double-cross hybrids and composite varieties, which are not allowed by using a heritable MS system due to fertility segregation. Thus, CIMS will greatly expand the utilizable range of OSR heterosis. These gametocides can also be used in a recurrent selection breeding program, with many advantages over using a GMS [32] because temporal CIMS can be achieved on any individual plant and at any cycle of selection without ever considering fertility segregation such as in a GMS [32]. In addition, an efficient SU or IM gametocide can be used by OSR breeders to replace the troublesome manual emasculation in sexual hybridizing.

## Conclusions

The application of most SUs and IMs had strong gametocidal effects as well as acceptable phytotoxic effects on OSR. This finding is of great importance for developing new functions for ALS-inhibiting herbicides. None of the tested TPs and PB resulted in MS, although these herbicides also obviously inhibited the activity of the ALS enzyme. This evidence suggests that inhibition of ALS activity may not always lead to plant MS, as previous studies have suggested, and ALS-inhibiting gametocide may also affect some other biological functions necessary for microspore development.

## Methods

### Plant materials

Seven OSR cultivars with different pedigrees and origins (Table 6), namely, Qin8C, Zhong9, Q5005, Zhe18, H15, Zheyou50, and M267, were used to evaluate the gametocidal effects. In addition, five Polima CMS lines, Zhong9-A, Sapphire-A, Q5005-A, Zheyou50-A, SP2-A, and the TMS line SP2S (Table 6), were used to study fertility regulation by gametocide. An inbred line, BC530, with a dominant trait of deeply divided leaves served as the male parent for cross-pollination because the presence of this trait in the derived F_1_ plants can indicate successful gene transfer from the male parent. The seed samples of these plant accessions (Table 6) are maintained by Dr. C.Y. Yu at Northwest A&F University.

**Table 6:** Pedigrees and origins of the plant accessions used in this study

Accession	Type	Pedigree	Origin^a^
Qin8C	Breeding line	Variety Qinyou8	AAS of Xianyang, China
Zhong9	Breeding line	Variety Zhongshuang9	ORI, CAAS, Hubei, China
Q5005	Breeding line	Variety Qianyou28	AAS of Guizhou, China
Zhe18	Breeding line	Variety Zheyou18	AAS of Zhejiang, China
H15	Breeding line	Variety Huyou15	AAS of Shanghai, China
Zheyou50	Breeding line	Variety Zheyou50	AAS of Zhejiang, China
M267	Breeding line	Variety Zheyou267	AAS of Zhejiang, China
Sapphire	Breeding line	Variety AV Sapphire	Australia
BC530	Breeding line	Variety Shaanyou16	NWAFU, China
SP2S	TGMS line	Reference [10]	NWAFU, China
SP2	Fertile near-isogenic line of SP2S	Reference [10]	NWAFU, China
Zhong9-A	Male sterile line	CMS maintained by Zhong9	NWAFU, China
Q5005-A	Male sterile line	CMS maintained by Q5005	NWAFU, China
Sapphire-A	Male sterile line	CMS maintained by Sapphire	NWAFU, China
Zheyou50-A	Male sterile line	CMS maintained by Zheyou50	NWAFU, China
SP2-A	Male sterile line	CMS maintained by SP2	NWAFU, China

^a^*AAS* Academy of Agricultural Science, *CAAS* China Academy of Agricultural Science, *NWAFU* Northwest A&F University, *ORI* Oilcrop Research Institute

### Screening of potential gametocide for OSR

The experiment was conducted mainly in the breeding nursery of Northwest A&F University, Yangling (longitude 108.07, latitude 34.28), China. The altitude of the experimental fields is 450 m, which is located in the temperature area. The long-term annual sum of rainfall is 600–800 mm, and the average daily temperature is 12.9 °C. The plants were grown in the experimental field in autumn, and gametocide treatment was carried out in the next spring. The field management during the experiment followed local practices, and weed control was performed manually. This experiment in the plant breeding nursery complied with the Regulations on Pesticide Administration in China.

The layout of the preliminary screening of new gametocides was a completely randomized design with three replications. In each plot, there were at least 45 seedlings of cultivar Qin8C. Each plot had three rows, and each row was two meters long with a row spacing of 40 cm. Twenty-seven herbicides (Table 1) were used in the study, including 20 SUs, two IMs (imazethapyr and imazamox), three TPs (flumetsulam, florasulam and penoxsulam), one PB (bispyribac-sodium), and one SC (flucarbazone-sodium). The trade names and chemical structures of all herbicides can be found in Additional file 1: Table S1. Based on the data from TBM and amidosulfuron, the empirical range of sublethal doses for each ALS-inhibiting herbicide was designed as 1, 2, 3, and 4% dilutions (higher doses were also used for the TP and PB members) of the recommended rate for weed control. All the doses/application rates in this paper refer to the active ingredient. An adjuvant saturate (Haina Co. Ltd., Qingdao, China) was added at approximately 0.1% concentration to enhance the adhesion of aqueous droplets on the leaf surface. Application was carried out using high pressure pump sprayers at the bolting stage of the plants, with the length of largest flower buds being ≤3 mm length (i.e., when the largest microspore is at the uninucleate stage). The solution volume was calibrated to approximately 2 ml per plant. The control plants were sprayed with water.

After herbicide treatment, the growth speed (stem elongation), flowering time, and flower size of the plants were investigated. Phytotoxicity was defined in this study as growth stop, upper leaf shedding, and flower bud wilt. The number of MS and fertile (including semisterile) plants were occasionally counted. The flower buds collected from ten random plants were fixed in a solution (ethanol/acetic acid 3:1 V/V) overnight and stored in 70% alcohol at 4 °C. The anthers of the flower buds were stained with acetocarmine solution and observed under a light microscope to estimate the pollen viability [19]. A viable pollen grain is oval shaped, and its cellular content is stained by acetocarmine, while a dead grain only has an empty shell that cannot be stained. The flower buds were processed with clearing agent methyl salicylate [20] to observe the pollen distribution in the anthers. The in vivo ALS activities of the flower buds treated with several classic herbicides were assayed as previously described [26].

### Validation of gametocidal effects of eight selected gametocides

An experiment was conducted to test the reaction of the six cultivars of semiwinter OSR, including Zhong9, Q5005, Zhe18, H15, Zheyou50, and M267, to two doses (modified from the suitable range in the above experiment) of eight selected gametocides in comparison with both the standard gametocide TBM and untreated control. The experimental plots were arranged in a split-plot design with three independent replicates. The six cultivars were placed in the main plot, and the gametocide treatments were in subplots. Each subplot contained four rows with 60 seedlings. The flowering time and the percentage of completely sterile plants in each plot were recorded. Ten plants were selected randomly and bagged to test the seed set under self-pollination. The seed-set rate from another ten plants under open/cross pollination was used to estimate the phytotoxic effect. The seed-set rates under self-pollination conditions and cross-pollination conditions were calculated with the following formula: percent seed-set rate = (number of seeds per treated plant / number of seeds per control plant) × 100.

To compare the optimal doses for seedlings with different plant heights or biomasses, we sowed the seeds of cultivar M267 in the field four times at five-day intervals and obtained four batches of seedlings with gradually reduced biomass due to the temperature decrease in the autumn. Every ten plants in these four batches of seedlings were sprayed with a series of solutions with a concentration gradient of 30, 40 … … 90, 100 mg/L chlorsulfuron, at a carrier volume of 5 ml/plant. The optimal dose, i.e., the dose resulting in nearly 100% MS and the lowest phytotoxicity, in each batch of seedlings was determined. Linear regression analysis was performed to evaluate the relationship between the optimal doses and plant biomass.

### Small-scale hybrid seed production and test of seed quality

A seed production trial was carried out in isolated cages to produce the necessary amount of experimental hybrid seed for yield trials. The maternal parents, Zhong9 and Q5005, were grown in two isolated plots that were covered by nylon mesh, along with the paternal BC530 with deep-divided leaves. Each isolated plot had a size of 6 by 8 m and contained 450 female seedlings. The plants were grown at the same density as that of the previous experiments, with a 2: 1 row ratio for each female to male parent. The female parents were exposed to 100 mg/ha chlorsulfuron when the main inflorescence of the bolting plants emerged from the upper leaf. Thirteen days later, the chlorsulfuron was applied again at a double dose. Cross-pollination was performed by manually shaking the male parent and dispersing its pollen grains to the style of the female parents. At maturity, seeds were collected from the treated female plants, and the seed germination rate was investigated in petri dishes containing moistened filter paper. The thousand-grain-weight of the hybrid seeds was determined, and then the seed samples were sown in the field. Two months later, the hybridity (percentage of true hybrid seeds) was estimated from the percentage of plants with the indicating trait of deeply divided leaves.

### Effect of chlorsulfuron exposure on unstable inherited MS lines

From OSR microspore development to the flowering period (early March to mid-April), the daytime temperature in Yangling continually increased from approximately 5 °C to 25 °C. Each set of four rows of the plants of the TMS SP2S and Polima CMS lines Zhong9-A, Q5005-A, Sapphire-A, Zheyou50-A, and SP2-A were treated with 100 mg/ha chlorsulfuron solution before the buds reached 3.5 mm long. Another set of plants treated with water served as the control. Ten randomly selected plants were bagged to test the selfed seed-set rate, and the rest were left to be cross-pollinated by the male parent BC530. The seed set of 10 sampled plants (under self-pollinated conditions as well as open-pollinated conditions) was investigated.

### Statistical analysis

The gametocidal effect and phytotoxicity of the chemicals were analyzed for variance using the statistical analysis package DPS 7.5 [33]. The means were tested by the least significant difference method at the *p* < 0.01 and *p* < 0.01 levels. To compare the effects of eight candidates to that of TBM, the effects of cultivars (main plots) and application doses (subplots) and the interactions between them were analyzed by using a split-plot model in DPS software. The results are shown in Additional file 1: Table S2.

## Supplementary information

**Additional file 1: Table S1.** The related information of herbicides used in the study**. Table S2.** ANOVA table for two-factor split plot designs.

## Data Availability

All the supporting data are included within the article and its additional files.

## Abbreviations

ALS: Acetolactate synthase
CIMS: Chemical induction of male sterility
CMS: Cytoplasmic male sterility
IM: Imidazolinone
MS: Male sterility
OSR: Oilseed rape
PB: Pyrimidinylbenzoate
SC: Sulfonylamino-carbonyltriazolinone
SU: Sulfonylurea
TBM: Tribenuron-methyl
TMS: Thermosensitive male sterility
TP: Triazolopyrimidine
