# Supplementary material for: Sublethal application of various sulfonylurea and imidazolinone herbicides favors outcrossing and hybrid seed production in oilseed rape
Source: BMC Plant Biol. 2020 Feb 11;20:69. doi: 10.1186/s12870-020-2278-9 (PMC7014721; doi:10.1186/s12870-020-2278-9)
Supplement: Supplementary file 1 — Additional file 1: Table S1. The related information of herbicides used in the study. Table S2. ANOVA table for two-factor split plot designs. [file 12870_2020_2278_MOESM1_ESM.doc]

**Table S1 The related information of herbicides used in the study ^#^**

| Chemical family | Herbicide | Company | Trade name |
| --- | --- | --- | --- |
| Sulfonylureas | Amidosulfuron | Bayer Crop Science (ex Aventis) | Gratil, Adret, HOE-75032 |
| Sulfonylureas | Bensulfuron-methyl | DuPont | Londax, DPX-F5384 |
| Sulfonylureas | Chlorimuron-ethyl | DuPont | Classic, DPX-F6025 |
| Sulfonylureas | Chlorsulfuron | DuPont | Glean, Telar, DPX-W4189 |
| Sulfonylureas | Ethametsulfuron-methyl | DuPont | Muster, DPX-A7881 |
| Sulfonylureas | Ethoxysulfuron | Bayer Crop Science (ex Aventis) | HOE 095404 |
| Sulfonylureas | Flazasulfuron | Syngenta (ex Zen.) | Shibagen, SL-160, OK-1166 |
| Sulfonylureas | Foramsulfuron | Bayer Crop Science (ex Aventis) | AEF-130360 |
| Sulfonylureas | Halosulfuron-methyl | Monsanto | Permit, Battalion, MON-12000 |
| Sulfonylureas | Iodosulfuron-methyl-sodium | Bayer Crop Science (ex Aventis) | AE F115008 |
| Sulfonylureas | Mesosulfuron-methyl | Bayer Crop Science (ex Aventis) | AE F-130060 |
| Sulfonylureas | Metsulfuron-methyl | DuPont | Ally, Allie, Escort, DPX-T6376 |
| Sulfonylureas | Nicosulfuron | DuPont | Accent, DPX-V 9360 |
| Sulfonylureas | Oxasulfuron | Syngenta (ex Nov.) | Expert, Dynam, CGA-277476 |
| Sulfonylureas | Primisulfuron-methyl | Syngenta (ex Nov.) | Beacon, Tell, CGA 136872 |
| Sulfonylureas | Pyrazosulfuron-ethyl | Nissan Chemical | Sirius, Agreen, NC-311 |
| Sulfonylureas | Rimsulfuron | DuPont | Titus, DPX-E 9636 |
| Sulfonylureas | Sulfometuron-methyl | DuPont | Oust, DPX-5648 |
| Sulfonylureas | Sulfosulfuron | Monsanto | MON-37500, Certainty, Maverick, Monitor, Monza, Outrider, Sundance |
| Sulfonylureas | Thifensulfuron-methyl | DuPont | Harmony, Pinacle, DPX-M6316 |
| Sulfonylureas | Tribenuron-methyl | DuPont | Express, Granstar, DPX-L5300 |
| Sulfonylureas | Triflusulfuron-methyl | DuPont | Safari, Upbeet, DPX-66037 |
| Imidazolinones | Imazamox | BASF (ex Am.Cy.) | Odyseey; Raptor; Sweeper; PESTANAL; AC299263 |
| Imidazolinones | Imazethapyr | BASF (ex Am.Cy.) | Pursuit, Pivot, AC 263499 |
| Triazolopyrimidines | Florasulam | Dow AgroSciences | Primus, DE-570 |
| Triazolopyrimidines | Flumetsulam | Dow AgroSciences | Broadstrike, Scorpion, DE 498 |
| Triazolopyrimidines | Penoxsulam | Dow AgroSciences | DE-638, Granite, Ricer, Viper, Bengala, Grasp |
| Pyrimidinylthiobenzoates | Bispyribac-sodium | Kumiai Chemical Industry Co. Ltd | Nominee, KIH-2023 |
| Sulfonylaminocarbonyltriazolinone | Flucarbazone-sodium | Arysta Life Science Corporation | MKH-6562, SJO-0498 |

# Heap, I. 2018. The international survey of herbicide resistant weeds. http://weedscience.org/summary/Herbicide.aspx#

**Table S2** ANOVA table for two-factor split plot designs

| Traits | Source of variation | Sum of squares | Degrees of freedom | Mean square | F | *p* |
| --- | --- | --- | --- | --- | --- | --- |
| Percentage of MS plant | Replication | 7.3831 | 2 | 3.6915 |  |  |
|  | A (cultivar) | 157.6634 | 5 | 31.5327 | 5.5170 | 0.0107 |
|  | Error (whole plot) | 57.1508 | 10 | 5.7151 |  |  |
|  | B (gametocide) | 166824.6174 | 18 | 9268.0343 | 2113.3280 | 0.0001 |
|  | AxB | 538.2631 | 90 | 5.9807 | 1.3640 | 0.0356 |
|  | Error (sub-plot) | 947.2715 | 216 | 4.3855 |  |  |
|  | Total | 168532.3494 | 341 |  |  |  |
| Selfing seedset rate | Replication | 2.4195 | 2 | 1.2098 |  |  |
|  | A (cultivar) | 9.8439 | 5 | 1.9688 | 2.4040 | 0.1114 |
|  | Error (whole plot) | 8.1885 | 10 | 0.8189 |  |  |
|  | B (gametocide) | 168976.8513 | 18 | 9387.6029 | 7437.9370 | 0.0001 |
|  | AxB | 138.2416 | 90 | 1.5360 | 1.2170 | 0.1264 |
|  | Error (sub-plot) | 272.6189 | 216 | 1.2621 |  |  |
|  | Total | 169408.1637 | 341 |  |  |  |
| Open-pollination seedset rate | Replication | 653.7962 | 2 | 326.8981 |  |  |
|  | A (cultivar) | 1849.3501 | 5 | 369.8700 | 5.8120 | 0.0090 |
|  | Error (whole plot) | 636.4173 | 10 | 63.6417 |  |  |
|  | B (gametocide) | 76271.0983 | 18 | 4237.2832 | 61.8560 | 0.0001 |
|  | AxB | 10735.3124 | 90 | 119.2812 | 1.7410 | 0.0006 |
|  | Error (sub-plot) | 14796.4447 | 216 | 68.5021 |  |  |
|  | Total | 104942.4189 | 341 |  |  |  |
